# Activation mechanism and activity of globupain, a thermostable C11 protease from the Arctic Mid-Ocean Ridge hydrothermal system

**DOI:** 10.3389/fmicb.2023.1199085

**Published:** 2023-06-19

**Authors:** Victoria Røyseth, Brianna M. Hurysz, Anna-Karina Kaczorowska, Sebastian Dorawa, Anita-Elin Fedøy, Hasan Arsın, Mateus Sá M. Serafim, Samuel A. Myers, Olesia Werbowy, Tadeusz Kaczorowski, Runar Stokke, Anthony J. O’Donoghue, Ida Helene Steen

**Affiliations:** ^1^Department of Biological Sciences, Center for Deep Sea Research, University of Bergen, Bergen, Norway; ^2^Skaggs School of Pharmacy and Pharmaceutical Sciences, University of California, San Diego, San Diego, CA, United States; ^3^Collection of Plasmids and Microorganisms | KPD, Faculty of Biology, University of Gdańsk, Gdańsk, Poland; ^4^Laboratory of Extremophiles Biology, Department of Microbiology, Faculty of Biology, University of Gdańsk, Gdańsk, Poland; ^5^Department of Microbiology, Institute of Biological Sciences, Federal University of Minas Gerais, Belo Horizonte, Minas Gerais, Brazil; ^6^La Jolla Institute for Immunology, La Jolla, CA, United States

**Keywords:** cysteine peptidase, clostripain, extracellular enzyme, metagenome bioprospecting, hydrothermal vent

## Abstract

Deep-sea hydrothermal vents offer unique habitats for heat tolerant enzymes with potential new enzymatic properties. Here, we present the novel C11 protease *globupain*, which was prospected from a metagenome-assembled genome of uncultivated *Archaeoglobales* sampled from the Soria Moria hydrothermal vent system located on the Arctic Mid-Ocean Ridge. Sequence comparisons against the MEROPS-MPRO database showed that globupain has the highest sequence identity to C11-like proteases present in human gut and intestinal bacteria. Successful recombinant expression in *Escherichia coli* of the wild-type zymogen and 13 mutant substitution variants allowed assessment of residues involved in maturation and activity of the enzyme. For activation, globupain required the addition of DTT and Ca^2+^. When activated, the 52kDa proenzyme was processed at K_137_ and K_144_ into a 12kDa light- and 32kDa heavy chain heterodimer. A structurally conserved H_132_/C_185_ catalytic dyad was responsible for the proteolytic activity, and the enzyme demonstrated the ability to activate *in-trans*. Globupain exhibited caseinolytic activity and showed a strong preference for arginine in the P1 position, with Boc-QAR-aminomethylcoumarin (AMC) as the best substrate out of a total of 17 fluorogenic AMC substrates tested. Globupain was thermostable (T_m activated enzyme_ = 94.51°C ± 0.09°C) with optimal activity at 75°C and pH 7.1. Characterization of globupain has expanded our knowledge of the catalytic properties and activation mechanisms of temperature tolerant marine C11 proteases. The unique combination of features such as elevated thermostability, activity at relatively low pH values, and ability to operate under high reducing conditions makes globupain a potential intriguing candidate for use in diverse industrial and biotechnology sectors.

## 1. Introduction

Proteases catalyze the hydrolysis of peptide bonds in proteins and are important in industrial applications (Gimenes et al., 2021). They are used in food and leather processing, as additives to detergents, as pharmaceuticals, and in biorefineries (Barzkar et al., 2018; García-Moyano et al., 2021). Proteases are among the most widely used enzymes globally, accounting for over 60 percent of all enzyme sales (Ward, 2011). Temperature-tolerant proteases offer the possibility for industrial processing at high temperatures by improving reaction rates, enhancing nongaseous reactant solubility, and reducing contamination by mesophiles (Kumar et al., 2000; Barzkar et al., 2018). Deep-sea hydrothermal vents sustain microorganisms at high temperatures (Kuwabara et al., 2007; Pikuta et al., 2007; Nunoura et al., 2008), making them an interesting starting point for the discovery of new thermostable proteases (Barzkar et al., 2018). Moreover, the increasing sequence diversity of encoded proteases revealed in hydrothermal vent microorganisms (Li et al., 2015; Dombrowski et al., 2018; Cheng et al., 2021) offers considerable potential for discovering new and novel proteases with optimized catalytic properties that may support future innovations.

Proteases are remarkably diverse in terms of activity and the nucleophilic residues that participate in hydrolysis (Rawlings and Bateman, 2019). Clostripain is a well-characterized endopeptidase originating from the bacterium *Clostridium histolyticum* (accession MER0000831) and is a member of enzyme family C11. Peptidases in this family are characterized by the presence of a catalytic cysteine-histidine dyad with a preference for hydrolyzing arginine and lysine bonds in the P1 position (Ogle and Tytell, 1953; Barrett and Rawlings, 1996; Labrou and Rigden, 2004). Clostripain-like proteases are synthesized as inactive zymogens that have various requirements for activation (Kembhavi et al., 1991; McLuskey et al., 2016). Some require divalent cations such as Ca^2+^ and/or reducing agents such as dithiothreitol (DTT) for activation and catalysis. Variance in the number of cleavage site(s) for activation is also observed, and in some cases, an amino acid linker peptide is removed (Gilles et al., 1979; Dargatz et al., 1993). Nevertheless, the resulting active peptidase will comprise of a light-and heavy chain making up a macromolecular active heterodimer. *In-trans* activation has been demonstrated in some, while others activate *in-cis*, reflecting the accessibility of cleavage sites to neighboring peptidase activity (Herrou et al., 2016; Roncase et al., 2017; González-Páez et al., 2019; Roncase et al., 2019).

This report presents a C11 protease called globupain, with “*globu*” representing its unclassified *Archaeoglobus* species origin and “*pain*” depicting it as a clostripain homolog. The type species, *Archaeoglobus fulgidus* (Stetter, 1988), of genus *Archaeoglobus*, was one of the first archaea to have its genome sequenced 25 years ago (Klenk et al., 1997). It has since served as a model thermophilic archaeon and has provided important information about archaeal DNA replication (Maisnier-Patin et al., 2002), DNA repair (Birkeland et al., 2002; Knævelsrud et al., 2010), thermostable enzymes (Madern et al., 2001; Steen et al., 2001) and enzymes of biotechnological relevance (Isupov et al., 2019; Palombarini et al., 2020). With globupain, we have discovered a novel archaeal clostripain-like protease with a complex activation mechanism. Its unique catalytic properties and high thermal stability makes globupain a promising candidate for industrial applications.

## 2. Materials and methods

### 2.1. Environmental sampling, DNA extraction, and sequencing

The Soria Moria vent field is part of the Jan Mayen vent fields (JMVFs), located at the southern part of the Mohns Ridge (Pedersen et al., 2005, 2010) in the Norwegian-Greenland Sea (71.2°N, 5.5°W). The end-member fluids of white smokers in the Soria Moria vent field have a pH of 4.1 and a concentration of hydrogen sulfide of 4.1 mmol kg^−1^ (Dahle et al., 2015). In June of 2011, an *in-situ* titanium incubator (Stokke et al., 2020) consisting of one chamber filled with 2 g of dried krill shells (Nofima, Bergen, Norway), mixed with grained flange rock material (Dahle et al., 2015), was deployed at ~30–35 cm below seafloor (blsf) in sediments at 716 m depth. The temperature was measured to be ~40°C and ~70°C at 20 and 30 cm blsf, respectively, indicating diffuse hydrothermal venting. The sample was recovered in July 2012, and the incubated material was immediately snap-frozen in liquid nitrogen and stored at −80°C. DNA was extracted with FastDNA™ SPIN Kit for Soil (MP Biomedicals, CA, United States) and sequenced at the Norwegian Sequencing Center in Oslo, NSC.^1^

### 2.2. Metagenomic assembly, binning, and annotation

For the primary metagenome, one plate of 454 GS FLX Titanium shotgun reads (average read length; 730 bp) was sequenced and assembled using the Newbler assembler v.2.8 (Roche, Basel, Switzerland) with a minimum identity of 96% over a minimum of 35 bases. In total, 0.9 million (75%) of the 454 raw reads were assembled into contigs resulting in 7448 contigs >500 bp and an N50 contig size of 10,887 bp. Open reading frame (ORF) predictions were made using Prodigal v2.60 (Hyatt et al., 2010) and screened against MEROPS (Rawlings et al., 2014; Release 9.13). Putative signal peptides were identified by SignalP v4.1 (Petersen et al., 2011). For the secondary metagenome, Illumina NovaSeq 150 bp paired-end reads were filtered and assembled using fastp v0.23.2 and MEGAHIT v1.2.9, respectively. Of the 290 million filtered reads, 90.8% mapped to the assembly using the bwa-mem aligner v.0.7.17 (Vasimuddin et al., 2019). Metagenome-assembled genomes (MAGs) were binned and refined using MetaWrap v1.3.2 (Uritskiy et al., 2018), which included the binning tools, MetaBat2 v2.12.1 (Kang et al., 2015, 2019), MaxBin2 v2.2.6 (Wu et al., 2016) and CONCOCT v1.0.0 (Alneberg et al., 2014). Contamination and completeness of the MAGs were assessed with CheckM v1.0.7 (Parks et al., 2015). Furthermore, the taxonomic classification of the globupain-associated MAG (INS_M23_B45) was performed using the GTDB toolkit v2.1.0 (Chaumeil et al., 2019, 2022) with the GTDB release 207_v2 (Parks et al., 2018, 2020, 2022). ORF predictions were made with Prodigal v2.6.0 (Hyatt et al., 2010) as part of the annotation workflow designed by Dombrowski et al., 2020. Cross-referencing the cloned globupain from the primary metagenome with the assembly from the secondary metagenome identified an ORF sharing 100% identity over 481 amino acids and 4 additional amino acids at the C-terminus (CFVD).

### 2.3. Sequence alignment and three-dimensional modeling

Sequence alignment was made using the ESPript 3.0 utility (Robert and Gouet, 2014). The amino acid sequences of distapain (MER0095672), clostripain (MER0095672), thetapain (MER0028004), and PmC11 (MER0199417) were retrieved from the MEROPS database (Rawlings et al., 2018). The alignment was based on NCBI BLAST+ sequence similarity search results using the blastp program (Madeira et al., 2022) with MEROPS-MPRO sequences.

The translated globupain DNA sequence (Supplementary Work Sheet 1) was submitted to AlphaFold (software version 2.1.1), available at NMRbox (Maciejewski et al., 2017),^2^ for prediction of a three-dimensional (3D) protein structure with atomic accuracy. AlphaFold (Jumper et al., 2021) modeled globupain structure was downloaded (Varadi et al., 2022), and the model’s alignment and analysis was assessed with PyMOL software (v0.99c; DeLano, 2006). Lastly, the modeled structure was compared to a previously published crystallized C11 structure (PDB ID: 4YEC; Roncase et al., 2017) template for comparison purposes.

### 2.4. Gene synthesis

**Figure 1:**
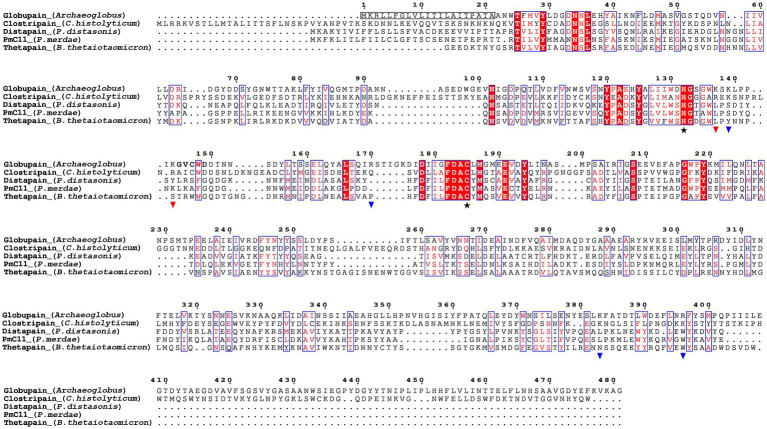
Sequence alignment of the C11 proteases globupain (*Archaeoglobus*), clostripain (*Clostridium histolyticum*), distapain (*Parabacteroides distasonis*), PmC11 (*Parabacteroides merdae*), and thetapain (*Bacteroides thetaiotaomicron*) by ESPript 3.0. Symbols depict results from site-directed mutagenesis of the globupain coding sequence; 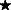, His/Cys catalytic dyad; 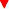, sites showing resistance against cleavage when the amino acid was mutated into alanine; 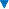, sites able to cleave when mutated into alanine. The detected N-terminal residues following activation are shown in bold.

Based on primary metagenome data, the globupain gene (GenBank accession OQ718499) was synthesized by GenScript (GenScript, NJ, United States) and codon-optimized for *Escherichia coli* expression (Supplementary Work Sheet 1). The gene was cloned (cloning site *Nde*I and *Xho*I) into pET-21A by GenScript, omitting the predicted 21 amino acid signal peptide (SignalP v6.0, Teufel et al., 2022). The resulting signal-free protein was extended with Met at the N-terminus, whereas the C-terminus was extended before the C-terminal hexahistidine tag (His tag) with Leu and Glu (LEHHHHHH). For identification of amino acids in the catalytic dyad and in maturation (Figure 1), targeted amino acid residues (Table 1) were substituted with Ala, and the respective coding genes were synthesized and cloned by GenScript as described for the wild-type (WT) globupain.

**Table 1:** Overview of mutation variants of globupain and their targeted function.

Position in globupain	Putative function
C_185_A	Catalytic dyad
H_132_A	Catalytic dyad
K_137_A	Self-activation
K_139_A	Self-activation
K_144_A	Self-activation
K_137_A/K_139_A	Self-activation
K_137_A/K_144_A	Self-activation
K_139_A/K_144_A	Self-activation
K_137_A/K_139_A/K_144_A	Self-activation
R_170_A	Self-activation
K_383_A	Cleavage C-terminal peptide
R_396_A	Cleavage C-terminal peptide
K_383_A/R_396_A	Cleavage C-terminal peptide

### 2.5. Protein production and purification

Expression plasmid of globupain and substitution variants were transformed into BL21-Gold (DE3) chemically competent *E. coli* cells (Agilent, TX, United States) using a heat-pulse manual supplied by the manufacturer. Cells were spread onto LB-agar plates supplemented with 100 μg/mL ampicillin and incubated at 37°C overnight. Pre-cultures were inoculated by picking one single colony and incubating at 37°C in LB media containing 100 μg/mL ampicillin with 190 rpm shaking overnight (Innova 44, New Brunswick Scientific, St Albans, United Kingdom). Expression cultures were inoculated with 5% of pre-culture in LB media with 100 μg/mL ampicillin at 37°C and 190 rpm. At OD_600_ of 0.6, the temperature was set to 20°C, and the culture was equilibrated for 30 min. Heterologous expression was induced by IPTG brought to 0.1 mM IPTG, followed by overnight incubation (20°C). Cells were harvested by centrifugation at 5,500 rpm for 15 min at 4°C (Allegra™ 21R Centrifuge, Beckman Coulter, CA, United States). Pellets were stored at −20°C.

For purification of globupain and substitution variants, cells were resuspended in lysis buffer (50 mM HEPES, pH 7.5, 300 mM NaCl, 0.25 mg/mL lysozyme, 10% glycerol), placed on ice for 30 min, and lysed by ultra-sonication (5 times with 30% amplitude, in intervals of 20 s on ice using the Vibra Cell with probe model CV188, Sonics and Materials INC, LT, United States). The lysate was clarified by centrifugation at 5,500 rpm for 20 min at 4°C (Allegra™ 21R Centrifuge, Beckman Coulter, CA, United States). The sample was then loaded into a HisTrap HP 5 mL column (Cytiva, Uppsala, Sweden) equilibrated with 20 mM HEPES, 500 mM NaCl, 25 mM imidazole, pH 7.5 with a flow rate of 1 mL/min. After elution with 20 mM HEPES, 500 mM NaCl, 500 mM imidazole, pH 7.5. fractions with the highest amount of enzyme were pooled and concentrated. The buffer was changed (20 mM HEPES, 150 mM NaCl, 0.1% CHAPS, pH 7.5) using Amicon® Ultra-15 centrifugal filter unit (Merck KGaA, Darmstadt, Germany) with a 30 K molecular weight cut-off. Approximately 1 mL of the concentrated enzyme preparations were purified by gel filtration using a GE 16/600 Superdex 200 pg. column (Cytiva, Uppsala, Sweden). Purified globupain and substitution variants were stored in 20 mM HEPES, 150 mM NaCl, 0.1% CHAPS, pH 7.5 at 4°C.

### 2.6. Maturation/activation

For activation of globupain and substitution variants (Table 1), purified enzyme (< 5 mg/mL) was incubated at 75°C for up to 4.5 h in 20 mM tri-sodium citrate dihydrate, 150 mM NaCl, pH 5.5 (at RT) with 2.5 mM DTT and 1 mM CaCl_2_, respectively (activation buffer). To investigate if globupain could *in-tran*s activate, 10 μg of activated WT globupain was mixed with 10 μg of inactive C_185_A variant. The number and size of cleavage products were assessed by visualization of protein bands on 8%–16% SurePAGE precast gels (GenScript) using MES SDS running buffer (GenScript) in a Bio-Rad Mini-PROTEAN Tetra Cell (BioRad, Hercules, CA, United States). For sample preparation, 4′ lithium dodecyl sulfate (LDS) sample buffer (GenScript) with 2-mercaptoethanol was mixed with the protein sample, followed by denaturing at 95°C for 10 min. Gels were stained with InstantBlue™ ultrafast protein stain (Abcam, Cambridge, United Kingdom), and the size of bands was indicated by broad multi-color pre-stained protein standard (GenScript). Edman sequencing was performed on a Shimadzu PPSQ-53A at the Iowa State University Protein Facility, United States.

### 2.7. Mass spectrometry sample preparation

Following staining with InstantBlue™ ultrafast protein stain (Abcam, Cambridge, United Kingdom), the gel bands (Supplementary Figure 10C) were excised and washed three times with 25 mM NH_4_HCO_3_ in 50% acetonitrile for 10 min each time. The gels were then dried completely in a Savant Speed Vac Plus AR (Thermo Fisher Scientific, MA, USA). A mixture of 10 mM TCEP and 25 mM iodoacetamide in 25 mM NH_4_HCO_3_ was added to cover the gel pieces and this reaction proceeded in the dark for 1 h. Gels were then washed with 25 mM NH_4_HCO_3_ and dehydrated with 25 mM NH_4_HCO_3_ in 50% acetonitrile. Samples were then dried in a Savant Speed Vac Plus AR (Thermo Fisher Scientific) before addition of 12.5 ng/uL trypsin in 25 mM NH_4_HCO_3_ for peptide digestion. Following a 10 min incubation at 4°C, the samples were covered in 25 mM NH_4_HCO_3_ and the digestion proceeded at 37°C for 20 h. The supernatant was then transferred to a clean tube and the remaining peptides were extracted from the gel by addition of 50% acetonitrile, 5% formic acid. The extracted digests were then dried and resuspended in 0.1% formic acid to prepare for C18 (CPI International) ZipTip desalting.C18 columns were washed with methanol and spun for 45 s at 3,500 × g. Columns were then cleaned and equilibrated with 50% acetonitrile, 0.1% formic acid and 0.1% formic acid in water, respectively. Samples were then loaded onto columns and spun for 2 min at 2,000 × g. Samples were washed with 0.1% formic acid and spun at 3,500 × g for 45 s. Peptides were eluted from C18 with 50% acetonitrile, 0.1% formic acid by spinning at 3,500 × g for 45 s. Samples were dried in a Savant Speed Vac Plus AR (Thermo Fisher Scientific) and stored at −80°C until they were prepared for mass spectrometry.

### 2.8. LC-MS/MS

Samples were redissolved in 0.1% formic acid prior to LC-MS/MS injection. Chromatography was performed as previously described on an Easy-nLC 1200 (Thermo Fisher Scientific; Myers et al., 2019). Mass spectrometry was performed on an Orbitrap Eclipse with ETD and PTCR (Thermo Fisher Scientific). The scan range was 350–1,800 m/z at a resolution of 60,000 with a 50 ms maximum injection time. The top 8 scans were selected for MS2. MS/MS spectra were analyzed in PEAKS Studio (v 8.5) software (Bioinformatics Solutions Inc.). MS2 data were searched against the combined *E. coli* (GCA_000022665.2) and globupain proteome (Supplementary Work Sheet 1). A precursor tolerance of 20 ppm and 0.01 Da was defined. Trypsin digestion was specified. The number of identified peptides were adjusted such that the false discovery rate was <1%. The data can be accessed on ProteomeXChange: PXD042411 or at ftp://massive.ucsd.edu/MSV000092007/.

### 2.9. Size-exclusion chromatography

Size-exclusion chromatography (SEC) analysis was performed using a Superdex 75 10/300 GL prepacked column connected to ÄKTA pure 25 chromatography system (GE Healthcare). The column was equilibrated with a 50 mM potassium phosphate buffer (pH 7.0), 150 mM NaCl and then loaded with a 500 μL sample of globupain protein (1 mg/mL). The flow rate of the run was adjusted to 0.5 mL/min, and the absorbance was measured at 280 nm (mAU, milli-absorbance units). For the experiment, the column was calibrated with proteins of known molecular weight: alcohol dehydrogenase (tetramer), 146,800; bovine serum albumin, 66,000; ovalbumin, 43,000; trypsin inhibitor, 22,000; and cytochrome C, 12,400 (Sigma-Aldrich, St. Louis, MO, United States). Dextran blue 2000 (Cytiva) was used to determine the column void volume.

### 2.10. Analytical ultracentrifugation

Sedimentation velocity experiments were performed in a Beckman-Coulter ProteomeLab XL-I analytical ultracentrifuge (Indianapolis, IN, United States), equipped with AN 60Ti 4-hole rotor and 12 mm path length, double-sector charcoal-Epon cells, loaded with 400 μL of samples and 410 μL of buffer (50 mM potassium phosphate buffer pH 7.0, 150 mM NaCl, and 1 mM EDTA). The experiments were conducted at 20°C and 50,000 rpm, using continuous scan mode and radial spacing of 0.003 cm. Scans were collected in absorbance, in 4 min intervals at 280 nm. Data were analyzed using the “Continuous c(s) distribution” model of the SEDFIT program (Schuck, 2000), with a confidence level (F-ratio) specified to 0.6. Biophysical parameters of the buffer: density (1,01395 g/cm^3^), and viscosity (1,030 mPa s), were measured at 20°C using Anton Paar DMA 5000 density meter and Lovis 2000 ME viscometer. Protein partial specific volume (V-bars) was estimated at 0.7309 mL/g using SEDNTERP software (version 1.10, Informer Technologies Inc., Dallas, TX, United States). The results were plotted using GUSSI graphical program (Brautigam, 2015).

### 2.11. Thermal stability analysis

Thermostability of the inactive globupain and its activated form were assayed by nanoscale differential scanning fluorimetry (nanoDSF). Measurements were performed with Prometheus NT.48 instrument (NanoTemper Technologies, München, Germany) and PR.ThermControl software using standard grade capillaries. Before measurement, the capillaries were sealed with a sealing paste according to the manufacturer recommendations. The results were further analyzed with PR.StabilityAnalysis software. Thermostability of globupain zymogen at 0.4 mg/mL concentration and its activated form were assayed in 20 mM tri-sodium citrate (pH 5.5) buffer with 150 mM NaCl and 20 mM HEPES buffer (pH 7.5), 500 mM NaCl with 25 mM imidazole, respectively. Melting temperature (T_m_) of proteins was determined by thermal unfolding with a temperature gradient between 20°C and 110°C at a ramp rate of 1°C/min. Thermal unfolding was measured by tryptophan and tyrosine fluorescence change at 330 and 350 nm emission wavelengths. All measurements were performed in triplicates.

### 2.12. Casein activity assays

The proteolytic activity of activated globupain and its substitution variants was assessed using the casein gelzan™ CM plate assay and the EnzChek™ Protease Assay Kit (Thermo Fisher Scientific, MA, USA), respectively. The gelzan™ CM plate assay was prepared by autoclaving 1.5% gelzan™ CM (Sigma-Aldrich) dissolved in 20 mM tri-sodium citrate (pH 5.5) buffer with 150 mM NaCl. The casein powder (Sigma-Aldrich) was dissolved in 20 mM tri-sodium citrate dihydrate (pH 5.5), 150 mM NaCl. NaOH was added until the casein was fully dissolved in the solution and then autoclaved at 115°C for 10 min. Casein was added to the gelzan™ CM solution at a final concentration of 1.0%. The casein gelzan™ CM solution was poured into sterile glass Petri dishes and set to harden. Wells were made by punching holes in the plates using an inverted sterile 1 mL pipet tip. To test for proteolytic activity, 60 μL of activated globupain at 0.7–1.0 mg/mL was added to wells and incubated overnight at 75°C. Clearance zones would indicate caseinolytic activity.

When the proteolytic activity was assessed using the EnzChek™ Protease Assay Kit (Thermo Fisher Scientific), 20 mM tri-sodium citrate (pH 5.5) buffer with 150 mM NaCl was used to dilute the 1.0 mg/mL stock solution of BODIPY FL casein to 10 μg/mL. An aliquot of the activated enzyme (0.15 μg) was then added to the reaction mixture (100 μL of total volume) comprising 12.5 μL of 10 μg/mL BODIPY FL casein working solution and 77.5 μL of 20 mM tri-sodium citrate (pH 5.5) buffer with 150 mM NaCl, 10 mM DTT, and 1 mM CaCl_2_. The caseinolytic activity was measured by running a time-resolved fluorescence read at 60°C, measuring fluorescence intensity every 20 s for 100 cycles. Fluorescence was measured with excitation wavelength 485 nm and emission wavelength 530 nm using an EnSpire™ 2300 Multilabel Reader (PerkinElmer, Turku, Finland). All measurements were done in triplicates and baseline corrected using GraphPad Prism 9.1.0.

### 2.13. Substrate screening

A total of 17 fluorogenic substrates containing a C-terminal 7-amino-4-methylcoumarin (AMC) reporter group were screened for hydrolysis by globupain. These substrates consisted of Ac-VLTK-AMC, Ac-VLGK-AMC, Ac-VLVK-AMC, Ac-IK-AMC, Ac-YK-AMC, Ac-LK-AMC, Ac-LETK-AMC, Ac-IETK-AMC, Ac-AEIK-AMC, Ac-AIK-AMC, Boc-LRR-AMC (R&D Systems S-300), N-Benzoyl-FVR-AMC (Bachem I-1080), z-RR-AMC (Sigma C5429), Ac-RLR-AMC (AdipoGen AG-CP3-0013), Boc-QAR-AMC (Peptide International 3,135-v), z-VVR-AMC (Peptide International 3,211-v), Pyr-RTKR-AMC (Peptide International 3,159-v) where the N-terminal blocking groups Ac, Boc, z, and Pyr, correspond to acetyl, tert-butyloxycarbonyl, benzyloxycarbonyl, and pyroglutamyl, respectively. Substrates containing K-AMC were synthesized by Dennis Wolan, The Scripps Research Institute, La Jolla, California and purified to >95%. All substrates were stored at −20°C as 10 mM stocks in DMSO. Substrates were diluted to 100 μM in 20 mM tri-sodium citrate dihydrate, 150 mM NaCl, 2.5 mM DTT, and 1 mM CaCl_2_, pH 5.5, and mixed 1:1 with globupain such that the final concentration in the assay was of 2.9 μg/mL enzyme and 50 μM substrate. Assays were performed in triplicate wells of a black 384-well plate (Thermo Fisher Scientific). Fluorescence was measured at 50°C over 1 h at excitation 360 nm and emission 460 nm on a BioTek Synergy HTX Multimode Reader (BioTek, Agilent, Tx, United States). The reaction rate was calculated as the maximum velocity over 12 sequential readings and means with standard errors were calculated. A Welch’s ANOVA and Brown Forsythe ANOVA were performed to calculate significances in GraphPad Prism 9.1.0. With Boc-QAR-AMC substrate, the Michaelis Menten kinetics was assessed at final concentrations ranging from 0 to 400 μM, and a Michaelis–Menten curve was fitted in GraphPad Prism 9.1.0 (Supplementary Figure 1).

### 2.14. Determining the pH-and temperature optimum

The pH optimum was determined using 5 nM globupain assayed against 50 μM Boc-QAR-AMC substrate in citrate phosphate buffer at various pH values. Buffers were made by mixing 0.2 M NaHPO_4_ and 0.1 M citric acid following the McIlvaine’s buffer system (McIlvaine, 1921) and pH was verified using a pH-meter. Samples were preincubated at 50°C for 10 min before fluorescence was measured. The optimum temperature for activity was assessed using Boc-QAR-AMC by incubating the enzyme and substrate at temperatures; 30°C, 40°C, 50°C, 60°C, 70°C, 80°C, 85°C, 90°C, 95°C, 100°C, 105°C, 110°C, 120°C, and 130°C in triplicate tubes in a final volume of 50 μL. The reaction temperatures were controlled using a digital dry bath (Thermo Fisher Scientific) with max temperature of 130°C set to the respective temperatures. After 10 min, the enzyme was inactivated by mixing 1:5 in 8 M urea. Samples were plated on a black 384-well plate (Thermo Fisher Scientific), and the total fluorescence was measured at excitation 360 nm and emission 460 nm. The data reported is the average RFU for each temperature with standard error. Gaussian distribution was fitted in GraphPad Prism 9.1.0. For the time-dependent loss of enzyme activity at pH 5.5 and pH 7.1, the enzyme activity (0.1 mg/mL) was measured at 60°C with the EnzChek™ Protease Assay Kit. All readings were done in triplicate reactions with the exception of duplicates for pH 7.1 at 120 min. Measurements were baseline corrected in GraphPad Prism 9.1.0. Statistical analyses were performed using RStudio 2022.07.1 + 554 “Spotted Wakerobin” release (RStudio, 2023).

### 2.15. Data availability

The native C11 globupain protease has been submitted to GenBank under the accession number OQ718499. The sequence is archived under BioProject PRJNA296938 and derived from the primary metagenomic assembly, BioSample SAMN04111445. The reconstructed *Archaeoglobus* genome, INS_M23_B45, has been archived under the BioSample SAMN33944460 derived from the secondary metagenome, BioSample SAMN33925184. This Whole Genome Shotgun project has been deposited at DDBJ/ENA/GenBank under the accession JARQZL000000000. The version described in this paper is version JARQZL010000000.

## 3. Results

### 3.1. Metagenomic globupain discovery

**Figure 2:**
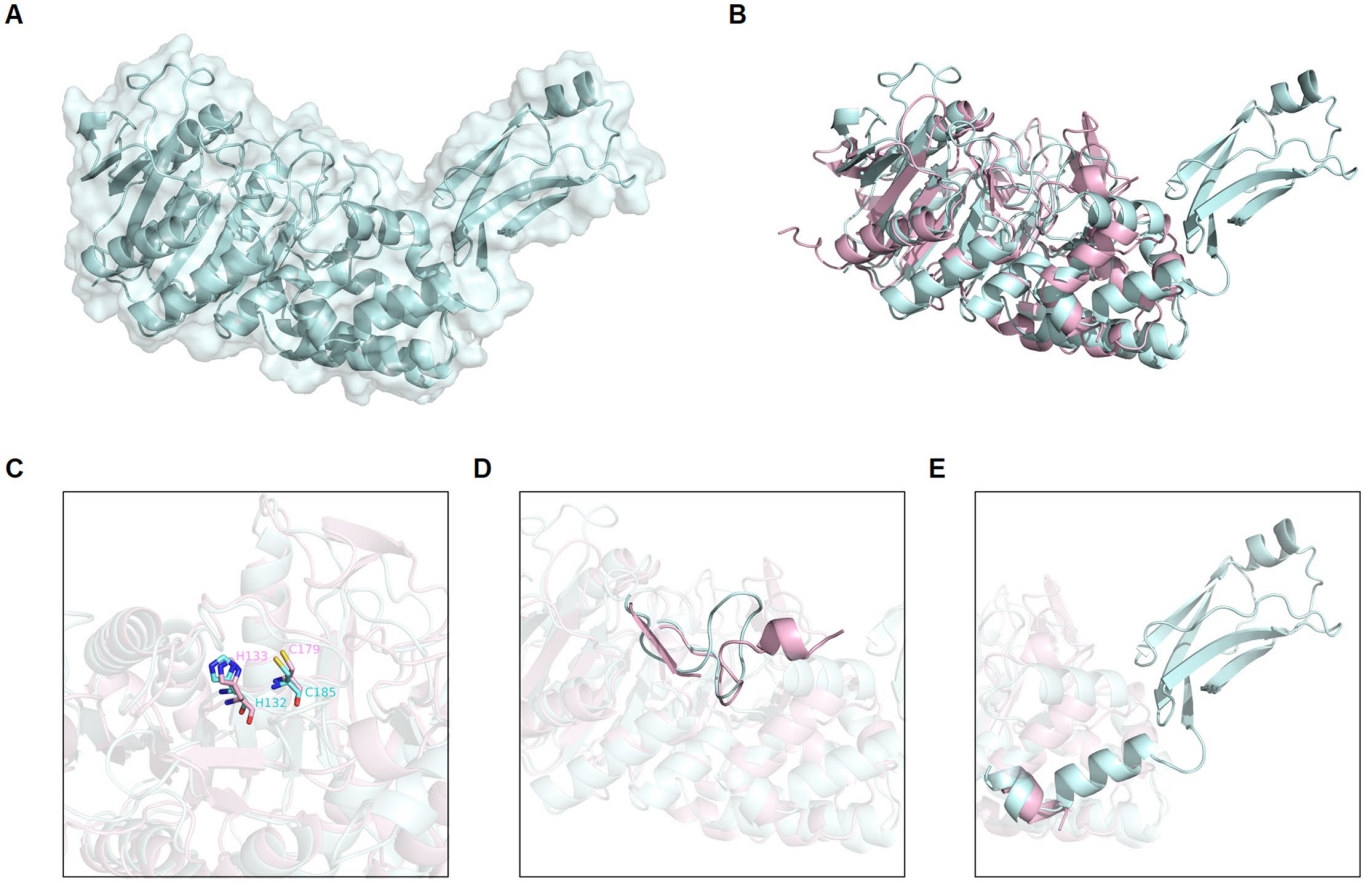
Globupain modeled structure predicted with AlphaFold in comparison to PmC11. **(A)** AlphaFold predicted structure of globupain is represented by cartoon with transparent surface (pale cyan). **(B)** AlphaFold of globupain compared to crystallized PmC11 (PDB ID: 4YEC) structure (light pink) in a 3D alignment showing their structural similarity. **(C)** Active site residues (e.g., His and Cys) are conserved among the two aligned structures. **(D)** Light-and heavy chain cleavage region is depicted for the modeled globupain superimposed to PmC11’s structure, as well as the **(E)** likely C-terminal cleavage region. Images were generated with PyMOL (v0.99c).

By conducting deep-sea hydrothermal *in situ* enrichments emended with targeted biomass, we have previously shown the induced shifts in community structure toward higher fractions of heterotrophic microorganisms (Stokke et al., 2020). Furthermore, *in silico* screening from derived metagenomes has shown a high potential for discovering novel enzymes (Fredriksen et al., 2019; Stepnov et al., 2019; Vuoristo et al., 2019; Arntzen et al., 2021). In the current study, a novel protease named globupain was identified from an *in situ* enriched metagenome and targeted for expression and characterization. The selected gene encoded a C11 protease and originated from a metagenome-assembled genome (MAG) classified as an uncharacterized genome within the genus *Archaeoglobus* (INS_M23_B45; SAMN33944460 INS_M23_B45; SAMN33944460). The putative polypeptide comprised 481 amino acids with a 21 amino acid N-terminal signal peptide (Figure 1). The estimated molecular mass after signal peptide removal was 52.0 kDa, and the pI was 4.2, as determined by the ProtParam tool (Gasteiger et al., 2005). The highest sequence identity scores against the MEROPS-MPRO database (Rawlings et al., 2016) were of the human gut and intestinal C11 members; clostripain of 23.5% (*C. histolyticum*), distapain of 27.3% (*Parabacteroides distasonis*), PmC11 of 24.2% (*Parabacteroides merdae*) and thetapain of 26.9% (*Bacteroides thetaiotaomicron*). Sequence alignments (Figure 1) indicated a conserved catalytic His/Cys dyad in globupain at positions 132 and 185, respectively. Moreover, in the globupain model obtained with AlphaFold (Figure 2A) deep learning-based algorithm (Jumper et al., 2021), structural similarities were observed between the predicted structure and the available PmC11 crystal structure (Figure 2B; PDB ID: 4YEC). The active site residues in PmC11 (e.g., D_177_), including catalytic H_133_ and C_179_, were conserved between both structures (Figure 2C). The residues which did not overlay well with PmC11 include an area between the known heavy-and light chain of PmC11 (Figure 2D) and a long C-terminal region (Figure 2E).

### 3.2. Globupain activation

**Figure 3:**
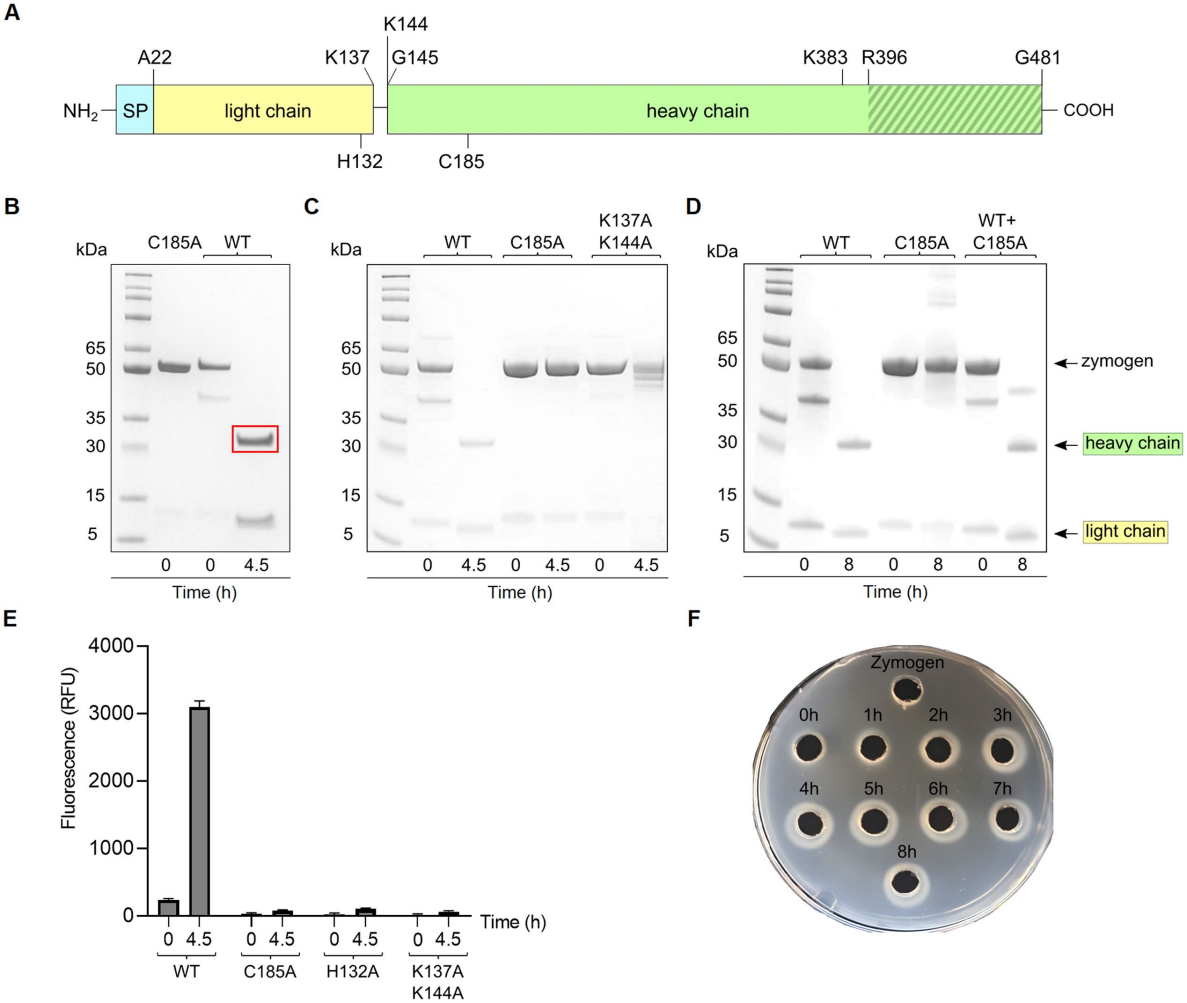
Activation of globupain. **(A)** Schematic representation of the primary structure of globupain. The enzyme was overproduced without the N-terminal 21 amino acid signal peptide (SP). The light chain (yellow) and the heavy chain (green) of the active heterodimer result from zymogen activation. Cleavage sites at K_137_ and K_144_ are shown. H_132_ and C_185_ of the catalytic dyad are indicated on the light-and heavy chain, respectively. The putative C-terminal region is indicated with gray stripes. Single and double mutation variants of K_383_ and R_396_ were tested for zymogen activation. **(B)** SDS-PAGE gel presentation of inactive C_185_A and wild-type (WT) of 52 kDa, respectively, and activation of WT into a 32 kDa heavy-and 12 kDa light chain when incubated at 75°C for 4.5 h in activation buffer. The region within the red rectangle was excised for N-terminal sequencing. **(C)** SDS-PAGE gel analysis shows that the C_185_A variant and K_137_A/K_144_A variant cannot process into a heterodimer. **(D)** SDS-PAGE image of WT, C_185_A and WT + C_185_A incubated at 75°C for 0 h and 8 h in activation buffer shows that the enzyme is able to *in-trans* activate. **(E)** When activated for 4.5 h at 75°C in activation buffer, globupain can cleave casein whereas mutation variants H_132_A, C_185_A and K_137_A/K_144_A showed no increase in fluorescence (RFU) when assayed with EnzChek™ Protease Assay Kit at 60°C. **(F)** Casein-gelzan™ plate showing globupain zymogen and clearance zones when activated at 75°C for 0–8 h in activation buffer.

Globupain and substitution variants were expressed as soluble proteins in *E. coli* BL21-Gold (DE3) cells, with almost 100% of the total recombinant protein as soluble enzyme (Supplementary Figures 2, 3). The purified WT enzyme (Supplementary Figure 4) was produced as an inactive zymogen. However, incubation at 75°C for 4.5 h (Supplementary Figure 5) in the activation buffer resulted in an active form of the C11 globupain. SDS-PAGE imaging showed that the 52 kDa zymogen was cleaved into a 32 kDa heavy chain and a 12 kDa light chain (Figures 3A,B), forming a heterodimer stabilized by noncovalent bonding. Oligomeric structure analysis performed by size-exclusion chromatography and analytical ultracentrifugation (AUC) revealed that globupain in zymogen form exists in solution as a homodimer (Supplementary Figures 6, 7). While the zymogen is inactive, the enzyme, after activation, can hydrolyze casein (Figures 3E,F). Globupain activation and its enzymatic activity, as in the case of other C11 proteases (Labrou and Rigden, 2004), depends on the presence of a His/Cys catalytic dyad in the primary protein sequence (H_132_/C_185_). H_132_ of the light chain is responsible for deprotonating the neighboring C_185_ in the heavy chain, which then promotes its nucleophilic attack on the substrate. For globupain, we found that substitution variant C_185_A generated by site-directed mutagenesis cannot be activated (Figure 3C). Also, no caseinolytic activity was observed for either H_132_A or C_185_A variants (Figure 3E).

Edman sequencing on a Shimadzu PPSQ-53A at the Iowa State University Protein Facility of the heavy chain revealed that the N-terminus consisted of G_145_VCWD; hence cleavage (*) occurred between K_144_ and G_145_ within the sequence LPPIK*GVCWD (Figure 1). To further evaluate globupain autoprocessing at this cleavage site, a K_144_A variant was constructed. Notably, processing of the zymogen into this variant’s heavy-and light chain still occurred with similar size of cleaved products as WT globupain, as visualized by SDS-PAGE (Supplementary Figure 8). To further assess if this result could be explained by cleavage after nearby Lys residues, 7 new variants were synthesized (Table 1; Supplementary Figure 8). Only the double (K_137_A/K_144_A) and triple (K_137_A/K_139_A/K_144_A) mutants failed to activate into the processed form (Figure 3C; Supplementary Figure 8) and remained catalytically inactive (Figure 3E), which altogether suggests that globupain can self-activate by cleavage after both K_137_ and K_144_ (Figure 1), respectively.

When mixing the WT zymogen with inactive C_185_A and performing the standard activation protocol, both the WT and C_185_A proteins were processed into the light-and heavy chain (Figure 3D). This finding demonstrates that globupain can activate *in-trans* and indicates that the sites for activation are exposed for cleavage by nearby proteases. Interestingly, the activation sites result in the removal of the unique region that poorly overlays with PmC11 (Figure 2D).

The combined molecular mass of the heavy-and light chain of activated globupain was determined to be 44 kDa, which, when compared to the 52.0 kDa zymogen (Figures 3A,B), indicates that additional autoprocessing occurs during activation. This discrepancy in molecular weight points to a likely cleavage in the C-terminal region, which the model supports (Figure 2E). Activated globupain (Supplementary Figure 9) failed to bind to the Ni^2+^ affinity column, and the C-terminal His tag of globupain was not detected on either light-or heavy chain (Supplementary Figure 10), altogether, revealing that a C-terminal fragment that contains the His tag was removed during autoprocessing. In-gel digest and subsequent proteomics of the 44 kDa protein band without C-terminal His tag upon activation (Supplementary Figure 10) revealed the most N-terminal and C-terminal tryptic peptides to be I_65_DGYDDSYGNWTTAK_79_L and F_384_ATDTLWDEFLNR_396_ (data can be found at ProteomeXChange: PXD042411 or ftp://massive.ucsd.edu/MSV000092007/). This corresponds to a 332 amino acid protein fragment with estimated Mw of 37.67 kDa using the ProtParam tool (Gasteiger et al., 2005). Although this proteomics study cannot clarify the exact cleavage location, it suggests that the site for C-terminal processing occurs at R_396_ or C-terminal of this site. From the sequence alignments (Figure 1) of globupain and several family C11 members, it is clear that R_396_ does not represent a conserved Arg cleavage site for the respective enzymes. Further, overlay of the PmC11 crystal structure (Figures 2B,E) and the modeled globupain structure suggest that the processing occurs in the non-conserved structural region of the two enzymes. Moreover, manual inspection of the primary sequence suggested that K_383_ and R_396_ might be the putative cleavage sites. However, each of the enzyme variants K_383_A, R_396_A, and K_383_A/R_396_A were still processed into the active form, and their C-terminal portion was removed (Supplementary Figure 11).

### 3.3. Substrate specificity (AMC) determination

**Figure 4:**
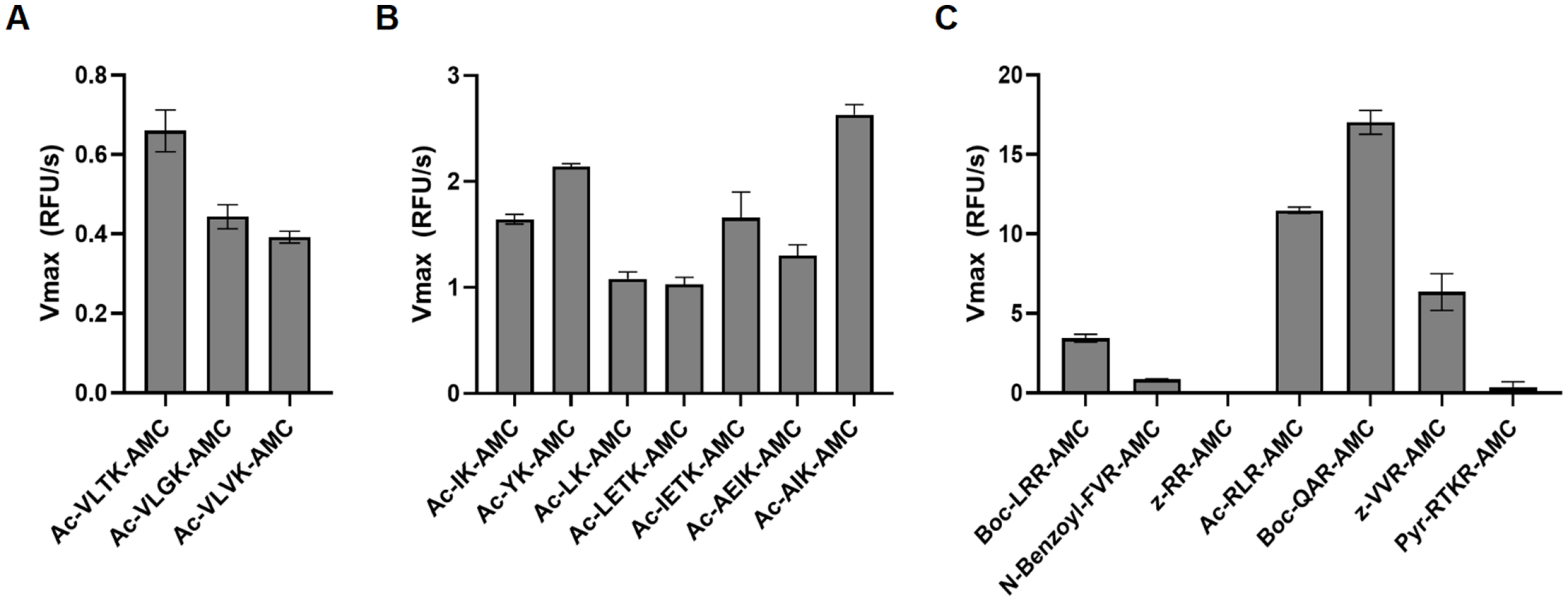
Substrate utilization by globupain. WT enzyme was assayed against 50 μM of each fluorescent substrate at 50°C, fluorescence was measured, and the rate of enzyme cleavage, V_max_, for each substrate is reported. **(A)** Globupain was assayed against the substrates initially designed for PmC11. **(B)** Globupain was assayed against 7 additional substrates with Lys at P1. **(C)** Globupain was assayed against 7 additional substrates with Arg at P1.

To quantify globupain activity in a microwell plate assay, the enzyme was incubated with three substrates that were previously developed for another clostripain-like C11 family member known as PmC11 (Roncase et al., 2017). This enzyme was encoded in the *P. merdae* genome. The substrates consisted of tetrapeptides (VLXK) with an N-terminal acetyl group (Ac) and a C-terminal AMC reporter group. These substrates were chosen as the P1 residue corresponds to the N-terminal auto-activation site of globupain. For PmC11, Ac-VLTK-AMC was most efficiently cleaved, followed by Ac-VLGK-AMC. However, the substitution of the P2 residue for a hydrophobic Val side chain ablated substrate turnover by PmC11. All three substrates were tested against globupain and found them to be cleaved at a similar rate (Figure 4A). This finding revealed that globupain has broader substrate specificity to PmC11 at the P2 position. Subsequently, 7 other substrates with Lys at P1 available in the laboratory were tested (Figure 4B). Globupain was able to cleave all substrates and cleaved Ac-AIK-AMC with highest efficiency. Interestingly, globupain cleaved each of the 7 new substrates more efficiently than the set of three initial substrates, which were based on the optimal substrate of PmC11 from *P. merdae*, indicating a distinct specificity to PmC11. The most statistically significant cleavage differences (*p* < 0.01) among the new 7 substrates occurred between Ac-AIK-AMC (the most efficient) and Ac-YK-AMC (least efficient). Activity with Ac-AIK-AMC was also statistically significantly increased (*p* < 0.01) compared to Ac-AEIK-AMC. We hypothesized that the broad enzymatic activity of globupain for degrading casein may be partially due to cleavage following a structurally similar amino acid, arginine. Therefore, fluorogenic substrates were examined with Arg as the P1 residue (Figure 4C). This screen of 7 additional substrates revealed that globupain cleaved substrates with even higher efficiency than the previous best with Lys at P1. However, some substrates, such as z-RR-AMC and Pyr-RTKR-AMC, showed minimal cleavage, indicating that globupain may favor non-polar amino acids at the P2 position. Out of the 17 AMC substrates that were tested in this study, it was clear that globupain had a strong preference for Arg in the P1 position, with Boc-QAR-AMC as the best substrate.

### 3.4. Thermal stability, optimal temperature, and pH

**Figure 5:**
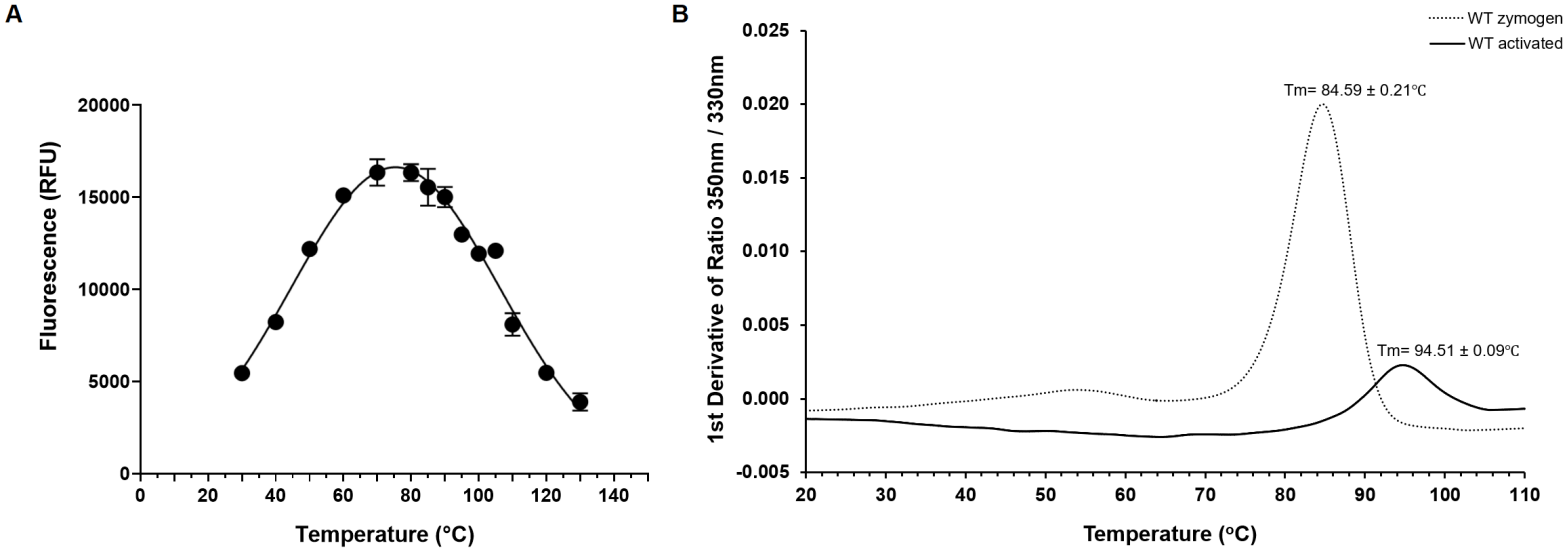
Thermoactivity and thermostability of WT globupain. **(A)** Optimal temperature of globupain activity determined by incubating globupain with the lead substrate, Boc-QAR-AMC, at various temperatures and inactivating with urea before measuring fluorescence which correlated to substrate cleavage. **(B)** Thermogram of zymogen and activated form of globupain. Y-axis represents the first derivative of fluorescence intensity ratio 350/330 nm measured by nanoDSF. T_m_ values are the mean and standard deviation from 3 replicate measurements.

**Figure 6:**
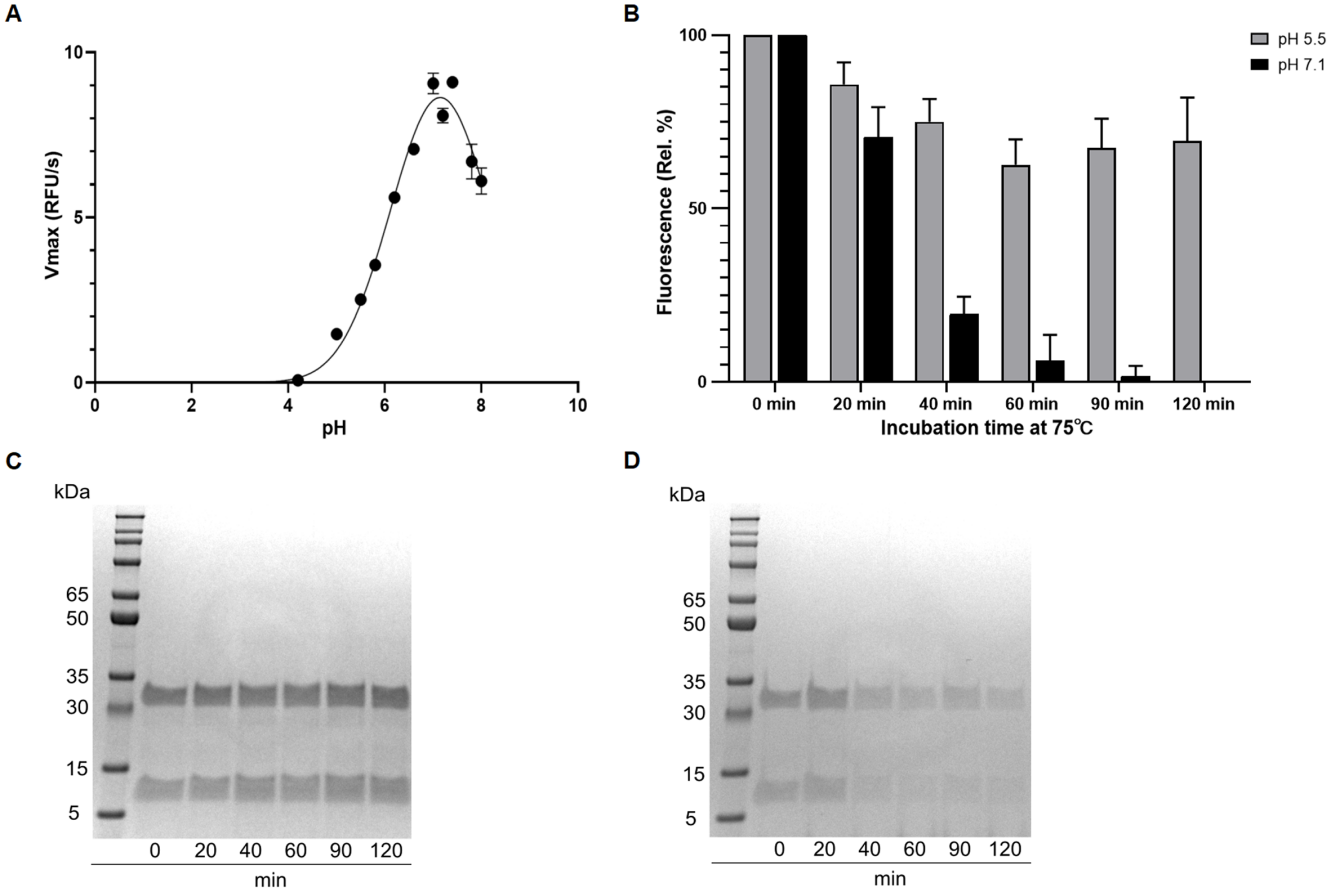
Effect of pH on activity and autolysis of globupain. **(A)** pH optimum resolved by assaying with the substrate Boc-QAR-AMC in buffers ranging from pH 2 to pH 8. Enzyme activity is shown as V_max_ for the different pH-values. **(B)** Time-dependent loss of enzyme activity at pH 5.5 and 7.1, respectively. Activity is shown as relative percent with standard deviations based on RFU measurements. **(C)** SDS-PAGE gel presentation reveals intact globupain after incubation at pH 5.5 whereas at pH 7.1 **(D)**, autolysis is observed, explaining the loss of activity in **(B)**.

Using the Boc-QAR-AMC substrate, the temperature optimum of globupain was determined to be 75.4°C ± 0.56°C and remained 90% active at 60°C and 90°C (Figure 5A). Thermal stability of inactive globupain as characterized by melting temperature, which indicates the point at which half the protein is unfolded was 84.59°C ± 0.21°C. The activated heterodimer’s melting temperature was 94.51°C ± 0.09°C (Figure 5B). Finally, the optimum pH of globupain using the Boc-QAR-AMC substrate was evaluated. The optimum pH for catalytic activity was calculated to be pH 7.1 (Figure 6A). Initial Analysis of Covariance (ANCOVA) indicated that there was a significant effect of pH on fluorescence after controlling for time, *F*(3,26) = 42.85, *p* < 0.05, R^2^ = 83.18%. A follow-up *post hoc* Tukey’s Honest Significance Difference Test (HSD) indicated that pH 7.1 had a stronger effect on decreasing RFU over time relative to the effect measured on RFU at pH 5.5, *p* < 0.05 (Figure 6B). Thus, while the optimum pH is higher than the pH used for activation, the enzyme was shown to be more stable against autolysis at pH 5.5 than at pH 7.1 (Figures 6C,D) which supported our use of pH 5.5 buffers for the biochemical characterization of globupain.

## 4. Discussion

In this study, we characterized the novel cysteine protease, globupain belonging to enzyme family C11. Globupain was prospected from metagenomic data assigned to an unclassified *Archaeoglobus* species from the Arctic Mid-Ocean Ridge vent fields. The enzyme was highly soluble, expressing at relatively high concentrations in *E. coli* (Supplementary Figure 4). Two protein bands (52 kDa and 40 kDa) with intact C-terminal His tag were visualized on SDS-PAGE gels after protein purification (Supplementary Figure 10). The zymogen of globupain is processed in the N-terminal region at K_137_ and K_144_ to yield a heavy-and light chain when exposed to activation conditions. Similar to clostripain from *C. histolyticum* (Kembhavi et al., 1991), globupain requires calcium and a reducing environment for activation (Supplementary Figure 5). This condition contrasts the C11 protease, PmC11 from *P. merdae*, which activates independently of calcium (McLuskey et al., 2016). When activated, the globupain enzyme cleaves off a C-terminal region which our proteomic analysis indicated that it was at R_396_ or C-terminal to this site. This kind of autoprocessing is not uncommon for C11 proteases; for example, activation of clostripain starts with a 23 amino acid pro-peptide removal (Dargatz et al., 1993). The two cut sites of globupain at K_137_ and K_144_, leads to the removal of a 7-amino acid linker sequence and the formation of a heterodimer consisting of a heavy-and light chain. For clostripain, a linker peptide is removed by cleavage at two Arg sites (Gilles et al., 1979; Dargatz et al., 1993). When activated, globupain showed the ability to *in-trans* activate and implied that the cut sites (K_137_ and K_144_) are exposed to proteolytic cleavage by neighboring proteases. This kind of activation is known to occur for several C11 enzymes such as thetapain (Roncase et al., 2019), fragipain (Herrou et al., 2016), and distapain (González-Páez et al., 2019) and contrasts PmC11 which activates only *in-cis* (Roncase et al., 2017). Globupain showed maximum activity at pH 7.1. This value is in the same range as known pH optima of PmC11 (pH 8.0), clostripain (pH 7.4–7.8), and thetapain (pH 7.4), respectively (Ogle and Tytell, 1953; Mitchell and Harrington, 1968; Roncase et al., 2017, 2019). However, globupain showed an optimum temperature of 75°C and matures into a heat tolerant enzyme, which allows it to function in its thermal environment (Dahle et al., 2015). The observed thermal properties are in line with the growth characteristics of cultivated species within the genus *Archaeoglobus* (Stetter, 1988; Burggraf et al., 1990; Huber et al., 1997; Mori et al., 2008; Steinsbu et al., 2010; Slobodkina et al., 2021) and enzymes characterized previously (Steen et al., 2001). Moreover, in comparison to well-characterized industrially relevant marine thermostable proteases, the thermal tolerance of globupain is superior to proteases sourced from marine *Bacillus* species and in the same range as of proteases from (hyper)thermophilic archaea (Barzkar et al., 2018).

Active clostripain-like proteases have been identified in marine sediment archaea (Lloyd et al., 2013). However, the highest sequence similarity scores of globupain using the MEROPS-MPRO database (Rawlings et al., 2016) were C11 proteases that originate from bacteria such as *C. histolyticum*, *P. distasonis*, *P. merdae, B. thetaiotaomicron* that have been found in the human intestinal microbiota (Salyers, 1984; Johnson et al., 1986; Franks et al., 1998). Some of these bacteria have been reported to cause disease and/or affect human health and have been studied to a greater extent (Salyers, 1984; Parracho et al., 2005; McLuskey et al., 2016; Roncase et al., 2017, 2019; Ezeji et al., 2021). This finding highlights the significance of acquiring greater knowledge of marine C11 proteases. Notably, all C11 proteases, including globupain, show a conserved His/Cys catalytic dyad by sequence alignment. Moreover, the catalytic residues were also conserved in the globupain model obtained with AlphaFold (Jumper et al., 2021). Finally, it was shown experimentally using site-directed mutagenesis that in globupain, H_132_ and C_185_ were critical for activation and activity. When assayed against several AMC substrates, the enzyme showed a clear preference for the substrate Boc-QAR-AMC. Preference for hydrolyzing Arg bonds in the P1 position is a known trait for C11 members (Ogle and Tytell, 1953; Labrou and Rigden, 2004). Globupain showed much lower activity against the Ac-VLTK-AMC substrate, which both PmC11 and thetapain hydrolyze efficiently (Roncase et al., 2017, 2019). This observation indicates that the substrate specificity may vary substantially between different C11 proteases despite having sequence and structural similarities around the active site. In conclusion, the revealed temperature tolerance and catalytic properties of globupain render it as a promising protease in diverse industrial and biotechnology sectors. Further studies focused on in-depth knowledge of the substrate specificity (O'Donoghue et al., 2012; Rohweder et al., 2023), effects of protease inhibitors, resistance to organic solvents and chemical denaturants may provide a deeper understanding of the applicability of globupain.

## Data availability statement

The datasets presented in this study are deposited in the NCBI online repository, under accession numbers PRJNA296938 (https://www.ncbi.nlm.nih.gov/bioproject/PRJNA296938), OQ718499 (https://www.ncbi.nlm.nih.gov/nuccore/OQ718499.1/), SAMN04111445 (https://www.ncbi.nlm.nih.gov/biosample/SAMN04111445/), and JARQZL000000000 (https://www.ncbi.nlm.nih.gov/nuccore/JARQZL000000000.1/).

## Author contributions

VR, AO’D, and IHS conceived the study. VR, BH, and IHS wrote the manuscript. VR, BH, A-KK, SD, A-EF, HA, MSMS, SM, OW, TK, and RS performed the experiments. All authors contributed to the article and approved the submitted version.

## Funding

This work was funded by the Research Council of Norway (RCN) through the Center for Excellence in Geobiology (grant #179560), the KG Jebsen Foundation, the Trond Mohn Foundation, the University of Bergen through the Centre for Deep Sea Research (grant # TMS2020TMT13), the CAPES Foundation (grant # 88887.595578/2020-00 and 88887.684031/2022-00), UFMG intramural funds, the RCN-funded DeepSeaQuence project (project #number 315427), and Norway Financial Mechanism through the National Science Center (Poland) GRIEG1 grant: UMO-2019/34/H/NZ2/00584. BH was funded by the UCSD Graduate Training Program in Cellular and Molecular Pharmacology through an institutional training grant from the National Institute of General Medical Sciences, T32 GM007752.

## Conflict of interest

The authors declare that the research was conducted in the absence of any commercial or financial relationships that could be construed as a potential conflict of interest.

## Publisher’s note

All claims expressed in this article are solely those of the authors and do not necessarily represent those of their affiliated organizations, or those of the publisher, the editors and the reviewers. Any product that may be evaluated in this article, or claim that may be made by its manufacturer, is not guaranteed or endorsed by the publisher.

## Footnotes

^1^ www.sequencing.uio.no

^2^ https://nmrbox.nmrhub.org/
